# Reports of Some Interesting Cases from the Surgical Clinics and Hospital of Dr. Van Langenbeck, of Berlin, Prussia

**Published:** 1872-03

**Authors:** A. W. Calhoun

**Affiliations:** Berlin, Prussia


### Foreign Correspondence.

### REPORTS OF SOME INTERESTING CASES FROM THE SURGICAL CLINICS AND HOSPITAL OF PROFESSOR VAN LANGENBECK, OF BERLIN, PRUSSIA.

BY A. W. CALHOUN, M. D., (NOW) OF BERLIN, PRUSSIA.

THE report and progress of cases after important surgical operations of different kinds, will, I imagine, not be unacceptable to those interested upon such subjects ; and so, from time to time, I shall do myself the pleasure of sending to your Journal a history of such, and their results, under the management of the first Surgeon in Europe. Almost every case treated in these Clinics is of vast interest and importance, as scarcely none but the graver operations are taken in hand by Prof. Langenbeck, and it is with some difficulty that I select from the large number amongst my notes, those I hope to be the most instructive.

In each one of the following cases Tracheotomy was performed, aside from the accompanying operations in two of them. In one the Tracheotomy was not completely finished, owing to the sudden entrance of relief.

CASE 1. *Resection of Right Superior Maxillary Bone for Fibro Sarcoma—Preliminary Tracheotomy—Rapid Recovery.*—A. D. A girl, aged 17, was brought into the Clinic Nov. 3d, 1871, with an enormous enlargement, occupying the entire right upper jaw. An examination showed the whole of the maxilla to be involved, with all the soft parts attached, in a diseased state. The overlying skin was sound, but quite thin from the long continued and increasing pressure from the tumor. Extending backwards, the swelling passed into the Pharynx, pressing strongly upon the soft Palate, and even loosening it from its bony connections, to a great extent—and farther forwards filled the right nasal cavity. Extending upwards, it involved the floor of the orbit, and threatened soon to seize upon the globe itself. The cause, or date of origin, is uncertain, but the growth is of several years standing, and has increased very rapidly for the last few months. Otherwise the girl is quite healthy, with no cancerous history in the family.

The disease was diagnosed Fibro Sarcoma, and an immediate operation decided upon, as there was great danger of perforation of the base of the skull—if this had not already occurred. Such operations are necessarily very long and bloody, and one of the great hindrances to their performance, is the danger of blood passing from the mouth into the lungs while under the influence of chloroform, thus producing suffocation. To avoid any trouble from this quarter, preliminary tracheotomy was performed. Chloroform was administered, and the infrathyroid incision made, and the different steps of the operation gone through with slowly, securing important vessels with the Torsion Forceps, as they were divided. After all hemorrhage had ceased, the trachea was opened, and a canula inserted, around which, for nearly its entire length, a thin elastic rubber sack fitted, and to the upper portion of which was attached a small rubber tube, extending outwards, through which the sack was inflated to the extent of filling the entire trachea around the canula, as if with a tampon, thus completely cutting off the passage of any blood into the lungs. To the canula itself was attached another tube, four or five feet in length, ending with a wire cap covered with flannel, over which chloroform was poured, and inhaled through tube and canula. By this means the patient was kept continually under the influence of the anæsthetic, without any interference to the operation.

*Operation.*—Made an incision extending from the inner side of inner canthus of the eye, downwards alongside the nose, inclining outwards, so as to pass the angle of the mouth ; turning outwards from the mouth, and slightly upwards, the incision was ended just above and in front of the tragus of the ear. Dissecting back this flap, the whole of the diseased and swollen bone was exposed. By the use of a fine saw, separated the entire sup. maxillary from its attachments, and removed it with the larger portion of the floor of the orbit. As the disease threatened to perforate the base of the skull, applied the actual cautery, and thoroughly cauterized all the neighboring parts. Immense hemorrhage followed the operation, which was finally checked by the cautery and ligatures upon the larger vessels. Brought the flaps together by an exceedingly large number of sutures, in order, as the professor said, to insure healing by first intention. The dressing consisted only of a cloth smeared with a simple salve and placed over the wound.

*Nov. 24th.*—Patient rested well during the night, under the influence of chloral hydrate ; vomited twice to-day, large quantities of blood that had oozed from the wounds and flowed into the stomach since the operation. No pain and but little fever. Ordered fluid diet, and the use of weak carbolic acid dressings over wounds.

*Nov.* 25*th.*—Slight fever; pulse 85; but no swelling of any consequence. Removed some of the sutures.

*Nov.* 27*th.*—Resting quietly, with every favorable symptom. Removed canula and brought tracheal wound together loosely with adhesive strips (immediately after the operation, the canula surrounded by the rubber sack, or tampon, was taken out, and a smaller one, without a sack, inserted in its stead.)

*Nov.* 28*th.*—The remaining sutures removed, and collodion strips placed across the wound. No pain, and very slight fever. To continue the same external applications and a nourishing fluid diet.

*Dec.* 18*th.*—In the interval from the 28th until to-day, the same simple treatment has been carried on, and to-day I find all the wounds entirely healed, with the exception of a few granulations upon the trachea. Previous to the operation the speech of the patient could scarcely be understood; now it is perfectly clear. The eye has never suffered in the least, although nearly the entire floor of the orbit was resected. The uvula and right half of the soft palate were also removed, but Prof. L. considers them of great importance in speech and deglutition, and advises their preservation in all cases where it is possible. She is improving daily in strength and flesh, and will be discharged in a few days.

*Jan.* 13*th*, 1872. Completely cured and to-day sent to her home in the country.

Case 2. *Resection of Right Superior Maxillary Bone for Epithelial Cancer—Preliminary Tracheotomy—Death.*—F. K., a Polander, aged 50 years presents himself (Dec. 19th, 1871) with a large open cancer upon right cheek, involving the outer coverings, as well as the deeper and bony structures. The disease has also seized upon the floor of the orbit, and a portion of the soft palate. In the extension of the tumor, this case is very similar to the one above reported. A very free and fœtid discharge flows from the open ulcer upon the jaw, in consequence of which the patient is much emaciated. The disease made its appearance in the early part of last summer, preceded by severe and continued pain, and in the last few months has increased rapidly in size, till it has now reached the extent described. Some of the symptoms of perforation of base of skull already exist. The age of patient, with his weakened condition, and the extent of the disease, makes the prognosis quite unfavorable; but clearly something must be done for the sufferer, and so an immediate operation is decided upon.

After giving chloroform, performed tracheotomy (infrathyroid), as in previous case, inserting similar canula, with tube attached, and surrounded by a rubber sack, which inflated, filled the trachea. (The working of this sack is fully described above.) Through tube and canula chloroform was contined till the operation was finished.

Commenced the incision just beneath the inner canthus of eye, and extending outwards underneath the lid to upper part of tragus of ear, directly downwards, to a point opposite the angle of the mouth, then forwards to the angle and upwards along the nose, meeting commencement of first incision. Sawing through the various connections, severed the entire superior maxillary with the diseased floor of the orbit, and the external diseased structures. The velum palati was kept in tact. Thoroughly cauterized all the parts with the "Ferrum Candens" to prevent a return. This left an enormous cavity upon the side of the face, almost one entire half having been removed, to fill which an important plastic operation was necessary. Dissecting the skin from the forehead, extending from right ear to beyond the middle line of frontal bone, and with its entire width (more than one-half the forehead), and turning it down over the cavity, stitched it in position by a large number of sutures, as in the former case. This flap will be well nourished, since it contains the temporal artery, yet there is for the first few days great danger of mortification in so large a transplanted portion of the skin.

The uncovered wound upon forehead was dressed with simple charpie, and will be allowed to heal by granulation. No blood passed into the trachea, owing to its being completely filled by the inflated rubber tampon, although the mouth was full during the entire operation. As soon as all bleeding ceased, removed the first canula inserted, and substituted a simple ordinary one. The same light dressings were used as before. Used stimulants rather freely, as patient was very much exhausted.

*Dec. 20th.*—Under large doses of chloral spent a tolerable night, and to-day has but little fever (pulse 90) or pain. No important swelling of the parts. Using internally strong fluid diet and stimulants—externally, carbolic acid dressings.

*Dec. 21st.*—Slept well during last night, but is restless to-day, and, withal, in not so good a condition as yesterday. To continue the same treatment.

*Dec. 22nd.*—Patient died to-day, and upon post mortem the disease was found to have extended alongside the trachea to the lung, where much pus was formed. Base of brain, with its membranes, were inflamed, and slightly implicated in the disease. The other organs were mostly free.

The great advantage, in such operations, of preliminary tracheotomy, and introduction of canula, is well demonstrated in these two, in some respects, similar cases. The surgeon is free from the great care and anxiety, lest blood pass into the lungs from the mouth, through the trachea, and produce suffocation—which is prevented by the inflated sack around the canula—and the patient himself is delivered from the terrible pains, by being kept, through the canula and tube, continually under chloroform, which otherwise would be a dangerous experiment.

CASE 3. *Perilaryngitis, producing all the death-threatening symptoms of Croup—Tracheotomy begun, and a Perilaryngeal Abscess opened, which relieved the necessity of continuing the operation.*—A child, aged 2 years, was brought into the Clinic Dec. 16th, with all of the worst symptoms of croup, and had struggled so violently for breath for the last few hours, as to be completely exhausted, and almost in a moribund state. Its first complaint was two days before, of pain around the throat, and to-day it has reached the above condition. The trachea showed but little inflammation, with no membranous deposit; yet there existed a tolerably distinct croupy cough. Upon and around the lower part of the trachea externally, there was slight swelling and *uncertain* fluctuation. It was thought that this swelling might press upon the trachea and produce the above dangerous symptoms; yet the case was quite obscure. At any rate, tracheotomy was clearly indicated, and possibly some external trouble might be found during the operation. Chloroform was given, and the lower operation for tracheotomy commenced. The different steps were gone through with slowly, checking the profuse hemorrhage from the enlarged and numerous vessels. A tenaculum was inserted into the bulging Perichondrium, which was raised and an incision corresponding to the external one made into it, when a large quantity of pus poured out, and continued to flow for a few minutes by pressure upon the sides of the neck, and from within the trachea outwards. The abscess was found to extend almost entirely around the trachea, between the cartilage and its membrane, both upwards and downwards for some distance. The respiration became immediately better, but not free, and the livid appearance of the lips and face partly disappeared. The operation was suspended, to be continued in an hour or two, in case the breathing did not further improve, and the other threatening appearances pass away. The wound was filled with charpie saturated in weak carbolic acid, and a loose bandage placed around the neck.

*Dec. 17th.*—Pus still discharging; the slight fever previously existing almost entirely disappeared. Breathing growing better, and child improving in all respects.

*Dec. 18th.*—Still improving; color of face and lips natural; no fever, and quite playful.

*Dec. 28th.*—Pus ceased several days back, the little patient completely cured, and to be discharged to-day from hospital. Prof. L. says such a case has never before come under his eye, and that, in all probability, necrosis of the cartilage of the larynx, in part, will take place, although this may require several months before such a result occurs.

Cases 4 and 5. *Tracheotomy upon two children in the last stages of Croup—Death of one in a few minutes, and the other two days after the Operation.*—The first brought into the Clinic Nov. 25th, and the other Dec. 7th. Both cases readily diagnosed as membranous croup, and in all respect quite similar. Each, apparently, was in a dying state when laid upon the table, and upon each the same operation (infrathyroid) was made, and canulas inserted. Profuse hemorrhage followed the incisions, from the vessels being immoderately enlarged through interrupted circulation. Tube-shaped pieces of false membrane were drawn through the laryngeal opening. The first case died in a few minutes after the operation; the second revived considerably, and some hopes were entertained of its recovery; but after two days it also died. Upon the latter the operation had the effect of prolonging life two days, for, without doubt, it could not have lived more than a few minutes, had it not been made. This short respite for the little sufferer shows what might have been the good result if its parents had submitted it to an operation several hours earlier.

The Supra and Infrathyroid incisions have been thoroughly tested in this hospital, and the statistics show that each possess advantages and disadvantages, which about counterbalance each other, but that cases frequently occur, to which one is better suited than the other.

It will be noticed that the operation for tracheotomy is easily and readily performed, and but little danger attached to it, and no doubt, were it made before a "*dernier resort*" demanded it, numbers of children, as well as adults, would be saved from the most horrible of deaths by suffocation, resulting from various causes.

Since writing the above, I have had to mourn the loss of a friend, from the same disease from which the two last cases died, and upon whom also the same operation was performed. He was the son of Prof. Hoffmann, Professor of Chemistry in the University of Berlin, and aged 23 years. After suffering for two days from Diphtheritic croup, and all other remedies having failed to check its progress, tracheotomy was performed by Prof. Langenbeck, with only temporary relief. In addition to the operation, immense quantities of oxygen were thrown into his lungs; but notwithstanding all treatment, he died two and a-half days after the first appearance of the disease.

Also has occurred in the Clinic another case of resection of the superior maxillary bone, in a woman aged 38, for Carcinoma occupying the left alveolar process, and extending into the antrum of highmore. Preliminary tracheotomy was performed as in the other two cases, and the same incisions made over the tumor, as in the first, and removed it, preserving intact the floor of the orbit, roof of the antrum, and the soft palate. The same dressings and treatment have been used throughout in this case, and now, several days (8) after the operation, every symptom is favorable, wounds are rapidly healing, and in all probability in a few days more she will be entirely cured.

*Jan. 19th*, '72.—Saw patient to-day. All sutures removed, and facial wound entirely healed.

One would suppose that after operations of such an extent, both the fever and pain would be great, but in each of these instances, the fever has been but slight, and scarcely any pain at all has existed.

Throughout the progress of these cases carbolic acid, in different proportions, has been used quite freely—and in all wounds about the head, and particularly upon the scalp, Prof. Langenbeck recommends it as having an especial favorable influence. This acid is used extensively in all the hospitals of Berlin, and with most pleasant results, upon almost all classes of wounds.

*Berlin, Jan. 20th*, 1872.

